# When does the co-evolution of technology and science overturn into technoscience?

**DOI:** 10.1007/s10202-011-0102-1

**Published:** 2011-11-29

**Authors:** Ulrich Fiedeler

**Affiliations:** Fraunhofer Institute for Solar Energy Systems (ISE), Heidenhofstraße 2, 79110 Freiburg, Germany

## Abstract

In this paper, the relations between science and technology, intervention and representation, the natural and the artificial are analysed on the background of the formation of modern science in the sixteenth century. Due to the fact that technique has been essential for modern science from its early beginning, modern science is characterised by a hybridisation of knowledge and intervention. The manipulation of nature in order to measure its properties has steadily increased until artificial things have been produced, such as laser beams, chemical compounds, elementary particles. Furthermore, the structural bracing of natural science, technological development, and industrial exploitation of nature go also back to the foundation of modern science. In order to strengthen the debate on technoscience against this background, the specific characteristics of technoscientific objects have to be clarified as have the specific characteristics of the social organisation of technoscience and its performance.

## Résumé

Cet article analyse les relations entre la science et la technologie, l’intervention et la représentation, le naturel et l’artificiel à la lumière de la formation de la science moderne au 16ème siècle. Compte tenu du fait que la technique a été un élément essentiel de la science moderne dès ses origines, cette dernière est caractérisée par une hybridisation de la connaissance et de l’intervention. La manipulation de la nature dans le but de mesurer ses propriétés a fortement augmenté jusqu’à ce que des choses artificielles soient produites comme les rayons laser, les composants chimiques, les particules élémentaires. De plus, l’entrecroisement structurel des sciences naturelles, du développement technologique et de l’exploitation industrielle de la nature remontent jusqu’à la fondation de la science moderne. A la lumière de ces éléments et dans le but de renforcer le débat sur la technoscience, les caractéristiques spécifiques des objets technoscientifiques doivent être clarifiées, de la même manière que les caractéristiques de l’organisation sociale de la technoscience et sa performance.

## Abstract (de)

In diesem Beitrag wird vor dem Hintergrund der Entstehung der modernen Naturwissenschaft im 16. Jahrhundert das Verhältnis von Naturwissenschaft und Technologie, von Intervention und Repräsentation, von Natürlichem und Widernatürlichem analysiert. Auf Grund der Tatsache, dass von Anfang an Technik ein wesentlicher Bestandteil der modernen Naturwissenschaft war, war Naturwissenschaft immer schon durch eine Hybridisierung von Wissen und Intervention charakterisiert. Die Manipulation der Natur zur selektiven Messung ihrer Eigenschaften hat stetig zugenommen bis künstliche Dinge wie chemische Verbindungen, LASER-Strahlen und Elementarteilchen produziert werden konnten. Darüber hinaus kann die strukturelle Verklammerung von Naturwissenschaft, technischem Fortschritt und industrieller Naturausbeutung ebenfalls bis zur Entstehung der modernen Naturwissenschaft zurückverfolgt werden. Zur Schärfung und Zuspitzung der Diskussion um die Technowissenschaften ist es angesichts der oben genannten historischen Zusammenhänge notwendig, die spezifischen Charakteristika technowissenschaftlicher Objekte zu klären und die spezifischen Charakteristika sozialer Dimensionen und Abläufe technowissenschaftlicher Prozesse herauszuarbeiten.

## Introduction

Since Thomas Kuhn (1976 [1962]) showed that progress in natural science is considerably dependent on extra-scientific aspects, such as personality, power, and culture, science studies have at least integrated the social dimensions of science into their analysis. As a result of these investigations, the influence of values, moral concepts and world views, of power and domination on the formation of notions, on scientific theories, and on the selection of research questions has become more and more evident and has questioned the ideal of science as the realm of objectivity and truth. Today, not only scholars of science and technology studies (STS) perceive science as a social process. Vice versa science has become a core element of modern societies in which essential systems, such as water and energy supply and transportation, rely on complex highly sophisticated technology. The importance of science for society is expressed most obviously by the idea that industrial societies are transforming into knowledge societies. These transitions affect not only the organisation of labour, production of goods and food, and almost all other aspects of daily life, but also the organisation of research and the production of knowledge.

The related developments concerning the organisation of research have been analysed by several scholars and have been discussed in terms such as, technoscience (Latour 1987), post-normal science (Funtowicz and Ravetz 1993), mode 2 (Gibbons et al. 1994; Nowotny et al. 2001), and triple-helix (Etzkowitz and Leydesdorff 1997). They have in common that the interactions between science, policy, and economy are increasing. Governments try to influence science with the help of research programmes in order to enable technological innovations and thus stimulate economic growth. But policy relies on scientific expertise in order to handle the complexity of modern societies. Science has to justify that it is worth its funding and tries to acquire private funding.

How the term technoscience relates to these general developments is still under discussion, whether it focuses on specific elements of this transition process or whether it represents a special analytical perspective. Along with this discussion, different concepts of technoscience exist. In her paper, Weber (2010b) has summarised the following different core elements which represent different approaches or at least foci within the concept of technoscience:

- “…the amalgamation of technologies with everyday life…” (p. 22)
- “…the implosion of traditional dualisms such as nature/culture, human/machine, subject/object and body/mind through the discourses and practices of contemporary science and technology.” (p. 19)
- “…the molecularisation of life…” (p. 26)
- “…the intimate coupling of human and machine, with the blurring of the boundaries between the human and the artificial and between body and mind.” (p. 26)
- “The emergence of a ‘new world order’ that comes not only with radical epistemological, ontological and socio-material changes but also with enormous socio-technical upheavals and restructuring of society and the symbolic order and fundamental changes in the nature of class, race and gender.” (p. 19)
- “Technoscience marks ‘a historical break–not with regard to socio-technical restructuring, but mainly with regard to the radical change of values of science and technoscience, respectively. … Technoscience is seen primarily as an entrepreneurial and pragmatic project in which technology assumes the leading role in developing innovative solutions for specific societal problems, as well as new markets.” (p. 21)

Technoscience as an historical break has been discussed in detail by Forman: “The abrupt reversal of culturally ascribed primacy in the science–technology relationship–namely, from the primacy of science relative to technology prior to circa 1980, to the primacy of technology relative to science since about that date–is proposed as a demarcator of postmodernity from modernity” (Forman 2007), abstract).

In general, two main perspectives that are combined in the discussion on technoscience can be seen. One perspective emphasises social aspects within the production of knowledge, such as power, domination, the role of specific actors, and profit; the other emphasises epistemic and more general aspects such as the convergence of science and technology, of representing and intervening, of understanding and performing, and of the natural and the artificial. I think that these different perspectives are related to different analytical approaches: the philosophical tradition of epistemology and the social perspective on technology (Weingart 2003, p. 41). One can argue that the latter is a further development of the former, but it seems to be that both approaches co-exist and have developed their own perspective (Greif 2002). “Philosophers care about justification, logic, reason, soundness and methodology. The historical circumstances of discovery the psychological quirks, the social interactions and the economic milieux are no professional concern of Popper or Carnap.” (Hacking 2008 [1983], p. 6)*.* On the other hand, as Weingart (2003, p. 82) pointed out, some social scientists reduce the production of knowledge merely to a process of social construction (see also Knorr Cetina 2007, p. 333). Following the discussion on technoscience of nanotechnology (e.g. Nordmann 2005; Fogelberg and Glimell 2003; Bensaude-Vincent 2004; Weber 2010b; Kastenhofer 2009), I had the impression that the blending of these different approaches leads to confusion within the argumentations. Notions such as pure science, disinterestedness and intervention have different meanings regarding the different analytical frames (philosophy vs. sociology), see subsection on “Purification”. Apart from these different approaches, I observe a further reason for confusion. Results that have been gained from the analysis of certain “technosciences” (e.g. life science, robotics) are often directly transferred to other technologies or scientific fields, such as physics, chemistry, or nanotechnology. But different fields have different “epistemic cultures” (Knorr-Cetina), especially concerning aspects of technoscience such as the “molecularisation of life” and the “blurring of the boundaries between the human and the artificial and between body and mind” (Weber 2010b, p. 26, 19); it is questionable whether these aspects could be observed in physics in the same manner as they can in life sciences.

From my perspective, it seems to be helpful to tease apart the different aspects which are discussed using the term technoscience in order to determine what exactly the term can be used to distinguish. The aim of this paper is to sharpen the notion of technoscience and to specify the different meanings of these aspects. In order to contribute to this challenge, I will analyse the relations between science and technology, intervention and representation, and the natural and the artificial. I will approach these subjects from the epistemological perspective and will concentrate on the role of technology in the knowledge production of modern science.

## Role of technology in modern science

The starting point of my analysis is the question, what, if at all, does nanotechnology characterise as technoscience and especially whether it marks a new relation between representation and intervention. An important element of the argumentation of Alfred Nordmann in his paper from 2005 is that the scanning tunnelling microscope (STM), one of the most prominent icons of nanotechnology,^1^ blurs the difference between observation and intervention.^2^ “With this instrument, the surface is strictly speaking not looked at but groped. Thus, we touch the subject of investigation with this instrument, we no longer passively observe it but actively intervene.” (Nordmann 2005, p. 7, transl. UF)

In order to understand the different aspects of the role of technology in science and to identify new aspects in today’s technoscience, I would like to look back into history. First I would like to turn attention to the origin of the term technology. The term originates from the Greek word techné which means skilfulness, the potential to produce something (Birnbacher 1998, p. 615). Techné means also to have the ability to reach intended aims. In other words, technique does not have a goal in itself but it serves an external purpose. “Technology is essentially a means of achieving arbitrary goals” (Birnbacher 1998, p. 613, transl. UF). Aristotle emphasised the aspect of production and creation in contrast to natural development and decay. Therefore, technique has its origin not in nature but in human activity. Techné describes the potential to produce something in a planned manner. It is “a creative acting which is correctly combined with rationality” (Aristoteles 1991, p. 235/[1140a9/10], transl. UF) or as we would say today technique is the “know how” to produce something (Krohn 1989, p. 21): the production of an artefact which is not naturally grown or taken from nature (like copper).

Here, we find the origin of the contradiction between nature and technology, the natural and the artificial: nature is the sphere of the given and natural grown technique is the sphere of human activity in order to reach an intended goal. In connection with this pair of opposites, there is a further original connotation of technique: applying technique means to outwit nature (Krohn 1989, p. 20). With technique, it is possible to mislead nature in order that it behaves differently than usual to serve man’s purposes. Therefore, Aristotle defines mechanics as outwitting nature or as acting against nature (Hermann 1991 [1980], p. 17), while the aim of philosophy is to understand nature (Krohn 1989, p. 20). Even in the early middle ages, techniques such as the Archimedean screw, which is used to lift water, were perceived as a trick to irritate nature and to bypass its usual behaviour (water does not flow upwards but downwards). This meaning could even be found alive in the term artificial with its root: artifice. This shows us that, very early on, technique could be perceived as a hybrid of knowledge (know how) and intervention.

## Birth of modern science

It is always a forced fit if one tries to find a certain historical data to mark a development which takes place over several tens or hundreds of years. There are some arguments to assign the birth of modern science to the work of Tycho Brahe, Johannes Kepler, Galileo Galilei, Frances Bacon, Isaac Newton (among others) (Schäfer 1999, p. 99; Störig 1992 [1950], p. 179). A number of societal, economic, and political developments, aetiologically relevant to the formation of modern science, happened in the sixteenth century in Europe (see Störig, p. 279). In the following, I will concentrate on developments which took place in the scientific realm and I will try to sketch the most important developments leading to the formation of modern science and their constituting elements.

During the sixteenth century, astronomy was the field in science which could be perceived as the pivotal field where traditional forms of seeking for knowledge were questioned and the principles of modern science were developed. The main reason was that observation (of the movement of the stars) and theory (world view) were no longer reconcilable, which led to the well-known Copernican Revolution.^3^

Building on the new paradigm set by Copernicus and on a huge set of data collected by Tycho Brahe^4^ during his life, Johannes Kepler has reformulated the planetary movements.^5^ Essentially, the principle he has derived from his work base on mathematics. He criticised the approach from Greek philosophers who have tried to explain nature by different qualitative forces. “Differently from them, he perceived nature quintessentially uniformly and all differences as only quantitative. But the reduction of qualitative differences to quantitative relations is the secret of the amazing success of science ‘Urbi material ibi geometeria’—where there is matter there is mathematics, Kepler was proclaiming and thus was formulating for the first time the ideal of cognition constitutive for all subsequent sciences.” (Störig 1992 [1950], p. 282, transl. UF).

Even more radical, Galileo Galilei has claimed that science must be based on quantitative mathematical descriptions of nature.^6^ The laws of nature were formulated by mathematics and did not aim to explain the essence of phenomena but aimed to describe its progression exactly. In other words, he did not ask why bodies fall (a question Aristotle tried to answer) but how they fall (Störig 1992 [1950], p. 283). Even though he came to his conclusions basically by thought experiment (Hermann 1991 [1980], p. 9), Galilei claims that objects never stop their movement unless a force is acting on them, that which we call today the “principle of inertia”. This is exactly contrary to Aristotle who claims that all objects have an inherent tendency to stop their movement. The essential difference is that it is no longer believed that it is in the “nature” of objects to stop their movement, but it is now believed that they are passive entities which are moved and stopped by forces according to natural laws.

Even though Galilei’s work is still based on analytical conclusion, it seems to be the essential step towards modern science. With this step, empirical science, with its hypothetico-deductive method, starts its advance and displaces the former dominant way of cognition by the metaphysical analysis of principles (Schäfer 1999, p. 99). Two important and constructive elements of modern science could even be observed in the work of Galilei: (1) abstraction and (2) experimentation.

### Abstraction

The first element is the conceptualisation of complex phenomena as a superposition of different effects. For example, in order to describe the movement of falling objects, this movement is composed of two antagonistic elements: attraction by the earth and friction by air. In “nature” the two effects could not be separated. It is an artificial conceptualisation to separate the two effects and describe them separately. Unrealistic and not observable concepts such as “ideal movement” (which is uniform and with no friction at all) are introduced. This disaggregation of different aspects from a complex phenomenon and the introduction of unrealistic, general and not observable phenomena and concepts—for example, inertia, velocity, acceleration, force, impact and so on—are the core elements of modern physics.

### Experimentation

The second element is the use of techniques to perform experimental measurements in order to test hypotheses and to describe natural processes with mathematical formulas (Schäfer 1999, p. 97). Technique is used to observe artificially separated phenomena and measure their progression. Galilei retarded the natural process of falling in order to be able to determine the mathematical relations between the abstract concepts which have been introduced: The distance (*x*) an object falls is proportional to the square of the time (*t*) taken for it to fall (*x* = ½*gt*^2^, *g* = const.). For that purpose, he used channelled ramps with different slopes. Galilei has “manipulated” falling in an artificial manner (Schäfer 1999, p. 67; Hermann 1991 [1980], p. 17). Therefore, Bacon concluded that the process of measuring comprises not just observation but an intervention in the natural process (Schäfer 1999, p. 105; Hermann 1991 [1980], p. 20). Experimentation means the creation of an artificial environment in order to observe aspects of nature which are naturally not observable (Schäfer 1999, p. 69).

We can conclude that the idea of the laboratory is to separate the object of investigation completely from its environment and to control every aspect of the process which is intended to be observed. On the practical level, complexity is reduced by adjusting and manipulating boundary conditions in order to observe artificial concepts and artificially separated effects.

Here, we see a new relation between cognition and technique. The renunciation of the traditional view of cognition, which was based on pure logical reasoning, has entailed the need for a new way of testing hypotheses: empirical experimentation. This conforms to the technical “manipulation” of nature in order to separate different artificially constructed phenomena. Now, technique is used to gain knowledge. This is related to a shift in the perception of the natural and the artificial. Technique is no longer seen as being antagonist to nature, or at least something that stands aside from nature, but is seen as a part of nature. Technique also obeys natural laws as everything else does (Hermann 1991 [1980], p. 18). This conforms to a shift in the conception of nature. While before nature was perceived as something which grows, is self-acting, and has an inherent telos, now it is perceived in an abstract manner, as complex machinery (Descartes, Laplace) organised by laws.

For my investigation on the question of if and how technoscience conquers the relation of science and technology, especially in epistemological respect, this is an important result. Therefore, here I would like to draw a provisional appraisal:

By definition, technology serves intended aims and is not used for its own sake. Within the heart of the notion, technique has a connotation as an opponent to nature: Technology is artificial. Furthermore, this connotation has the meaning of outwitting or misleading nature in order to accomplish human goals. The formation of modern science marks a turning point in the production of knowledge. While previously cognition was pursued by pure reasoning, in modern science the application of technology is an essential part of the production of knowledge.

Modern science is characterised by:

- Disaggregation of different aspects (e.g. gravitation, drag) of a complex phenomenon (the falling of an object through the air) and introduction of unobservable concepts such as force, velocity, inertia and impact.
- The formulation of the laws of nature which are valid for every kind of matter. This is achieved by abstraction from the concrete object as well as from the concrete observer and related contingencies such as time and location.
- Not asking why things happened but how things happened and trying to describe these processes quantitatively by mathematics.
- Gaining knowledge not by intuition or pure logical conclusions but by observing nature in artificial circumstances: the experimental set up is characterised by the use of technology for manipulating natural processes in order to observe and determine the progression of processes or specific aspects.

### Modern science as applied science

I would like to cite a further aspect of technoscience which could be traced back to the foundation of modern science: the change in direction from the ideal of research for its own sake (to gain knowledge) to the idea of understanding nature to be able to manipulate nature in order to serve human purposes. Francis Bacon is perhaps the most prominent person who has proclaimed that the purpose of science is to raise the living conditions of humans^7^ (Schäfer 1999, p. 95–96, 100, 102, 105f). The change to empiric knowledge production is the precondition of a purpose-driven science. Schäfer emphasises that, due to Bacon (and other protagonists of that time, for example Descartes, Hobbes), modern science is rooted in a structural bracing of natural science, technological development, and the industrial exploitation of nature (Schäfer 1999, p. 97). Today, we would call this kind of purpose-driven cognition applied science, which found its aim outside of the process of the knowledge production, while pure science only seeks to increase knowledge.^8^ Here, we have a further connection to an aspect which is discussed with the term technoscience: the relation of science, technology and the increase of economic wealth and progress.^9^

### Semiconductors

In the following, I would like to discuss two more fields of physics in order to analyse the relation of intervention and representation. The first field is semiconductor physics which is a part of solid-state physics. The interesting point with respect to our analysis is that fundamental new insights in semiconductor physics have been gained since the development of the knowledge for growing pure crystals (Wagemann 1992, p. 473f). Similar to the problem discussed above, the separation of the antagonistic forces—acceleration by gravitation and drag by air—in semiconductor physics, it is necessary to investigate an idealised system, for example a perfect crystal, in order to be able to test theoretical models empirically.

Thus, at the beginning, the driving force was not only the application of semiconductor physics but also the development and proof of the theoretical models and their mathematical descriptions.^10^ In order to do this, it was necessary to build extraordinary pure crystals—something which does not exist in nature and requires tremendous effort. Crystals which show semiconductor behaviour are extremely artificial. In semiconductor physics, we can perceive several aspects which are characteristic of technoscience:

- It is based on the production of extraordinary artificial objects which are the subject of investigation.
- Technique is essential to produce semiconductors with sufficient purity.
- Purity is an important precondition for cognition.
- Semiconductor physics is strongly application oriented.

### Elementary particles

The second example is particle physics. This field is characterised by the production of entities which are as artificial as semiconductors. In addition, they cannot be observed directly because they decay immediately after they have been produced.^11^ Incredible, highly sophisticated machines with ultra high vacuums are necessary to produce them, and similar complex machines are necessary to observe them. Ontologically it is unclear whether they exist (or have existed before the decay) at all. It is obvious that these particles and the characteristics that are attributed to them like “spin”, “parity”, “flavour” and “colour” are constructions which may correspond to aspects of nature but which are entirely dependent on theory and could not be observed or denoted if the theory were different. But these characteristics are helpful in order to setup hypotheses which could be experimentally verified or falsified. However, very differently to semiconductor physics, no serious idea exists as to how this kind of technology could be applied in order to solve “real-world” problems or to increase welfare and raise general wealth. Regarding technoscience, we can conclude that as with semiconductor physics, particle physics:

- Is based on the production of extraordinary artificial objects which are the subject of investigation.
- Technique is more than essential for the production of these particles.
- In a certain sense, purity (an ultra high vacuum) is also essential to observe these objects.

But in contrasts to semiconductor physics, this research is not application oriented.

## Discussion

If we compare these findings with the subjects that are discussed in relation to technoscience, we can conclude that, in the foundation of modern science, we find essential elements of these subjects. These elements are discussed in the following sections.

### Nature, technology and the artificial

The term technosciences is used to analyse the relationship between the artificial and the natural which have been changed due to the new organisation and orientation of modern-day science. Here, technology is the essential factor that determines and permanently shifts the relation between the artificial and the natural. While this seems to be obvious, if we are considering how strongly our every day life is influenced by technology, the role of technology and thus the shift of the boundary between the natural and the artificial in modern science is also obvious but less clear. The first part of this paper has shown that the use of technique in science, especially in order to manipulate nature, has been characteristic since its beginning in the sixteenth century. By introducing the experiment as the main principle for cognition in natural science, nature is no more observed as one finds it but it is manipulated in order to show specific aspects which are only observable in an artificial environment.^12^ This creation of artificial objects might have come to its temporary peak with the creation of elementary particles in high-energy physics. But these particles are, in principle, in line with objects such as electrical current, microwaves, laser beams, elements of the periodic table and chemical compounds.

Along with the development of modern science, we can observe a change in the perception of the relation of nature and technique. Technique is no more seen as contradictory to nature. Technique has to follow the same laws as do processes in nature and vice versa. But while the interconnectedness of science and technology has changed continuously up to now, with the foundation of modern science during the sixteenth century, an epochal break could be observed regarding the perception of the relation of nature and technology. While previously nature was perceived as something which grows, which is self-acting, and has an inherent telos, now nature is perceived in an abstract manner and as complex machinery which is organised by laws. This concept was even extended to living beings and the human body (Descartes 1986 [1641], p. 201[83/84). It has led to enormous progress in cognition; thus, one can even explain how specific chemical agents influence mental states. It has contributed to the mechanisation of daily life (up to the most intimate personal relations). One might assign the climax of this concept to the 1960s when man was exhilarated by space flight and the landing of the first man on the moon. This cognition-guiding concept has experienced its biggest crises due to ecological and technical catastrophes which have resulted in insights into the complexity of ecological systems (Carson 1962) and the uncontrollability of big technologies (Luhmann 1991; Douglas and Wildavsky 1993; Jonas 1987; Schäfer 1999). But, in the framework of nanotechnology, this concept seems to have experienced a renaissance. In 1986, Drexler conceptually designated nanotechnology as molecular engineering (Drexler 1986). Biotechnological objects, including genetic engineering, are perceived as “soft machines” (Jones 2004) and are paradigmatic for synthetic biology (Deplazes and Huppenbauer 2009).

### Intervention and representation

In the previous section, it was made clear that technology is the pivotal point with respect to the relation of the natural and the artificial and it is, at the same time, the core element of modern science. It is often stated that nanotechnology will revolutionise the potential of technology. It will enable man to “shape the world atom by atom”. If one is examining the technical world^13^ which is assigned to nanotechnology, its revolutionary potential is hard to comprehend (see Schummer 2009a, b). Nanotechnology offers no new tools and techniques which are essentially different to techniques used to generate artificial objects like semiconductors, chemical compounds and elementary particles. Here, I would like to return to the initial question: Does the STM mark a specific relation between intervention and representation? Compared with the abovementioned examples relating to the STM, we cannot determine new fundamental aspects, such as the inability to abstract the observable fact from the artefact which could not be observed in former experimental praxis.^14^ The fact that information about a surface is generated by scratching the surface with a needle is not an expression of a new kind of mix of manipulation and representation. It is one step in the continuous refinement of the use of technology within experimentation. The use of technology within experimentation is one of the constituencies of modern science but not new or specific to nanotechnology.^15^ Continuous development would become more clear if one compares this experimental praxis with other measurement tools, such as, among others, the scanning electron microscope (SEM), which was invented in the early 1930s or with X-ray diffraction (XRD) which was used for the first time in 1912 (Luger 1992, p. 110).

What I would like to emphasise here is that in physics, the fundamental concepts regarding the relation between the natural and the artificial were set up during the development of modern science in the sixteenth century. Since then, techniques have become more and more sophisticated and more and more important for natural science. But this is a continuous process which is not finished nor has it, at least in physics, entered a new qualitative level.^16^ This might be different in life science. Techniques, such as genetic engineering, in vitro fertilisation and cloning, might introduce new qualities into the relationship between the natural and the artificial. For me, it seems to be plausible that hybrids such as the onco-mouse are extraordinary examples of how technical intervention is extended into living beings. Here, we find a mixture of the naturally grown and the artificially produced which might be significantly different from previous manipulations produced by breeding which leads to high-output cows or pigs with extra vertebra. Another field where the traditional concepts of the natural and the artificial are contested could be found when it comes to human nature and the technical interventions into the human body such as doping or to change the sex or for cosmetic surgery and other kinds of human enhancement^17^ (Barkhaus and Fleig 2002a; Habermas 2001).

### Purification: pure science, applied science and disinterestedness

Another essential complex that is discussed with the help of the term technoscience is the role of interests within the production of knowledge. Some colleagues see the *differenzia specifica* of technoscience in the orientation under which it is performed: technoscience is purpose driven, in contrast to science which is curiosity driven;“Basic technoscience research is dedicated to the acquisition of basic capabilities of visualisation, manipulation, modelling and control and is not dedicated to the advance of the Enlightenment by way of truth seeking or the criticism of prejudice and superstition” (Nordmann 2010, p. 7/8; see also Nordmann 2004, p. 59/60; Weber 2010a, p. 12/13); or in the words of Forman “the primacy of technology relative to science instead of the primacy of science relative to technology” (Forman 2007, p. 9) which mark a change in valuation. (But then we have to acknowledge that, e.g. elementary particles are no technoscientific objects).

In the introduction, I wrote that the different perspectives on cognition—philosophical and sociological—are related with different connotations of terms like pure science, disinterestedness, and intervention. Therefore, I would like to shed light on the different meanings of the terms “pure” and the “work of purification” (Nordmann 2010, p. 7 footnote 2). The following meanings can be distinguished:

*1.*: *Free of experience* Science could be pure because it is not based on empiricism. Pure science seeks idealised fundamental principles. This is related to the original concept of Greek philosophy and has been re-evaluated by Kant when he tried to investigate how synthetic conclusions a priori would be possible.^18^ The superiority of theory^19^ over praxis has its roots in Greek philosophy (Schnädelbach 1998, p. 40). Cognition has been considered to be of value in itself (Schäfer 1999, p. 102) and has been evaluated more highly as craft. These moral concepts were present until the last century and can be observed even today when humanities are perceived as sublime and engineering as ordinary craft (Birnbacher 1998, p. 606; Weingart 2001, p. 60). Exactly the re-evaluation of the relation of theory and praxis in science, which Forman dates to the 1980s, is that which he identifies as the turn of science into technoscience. In a similar vein is the idea that pure science (theory) is not made impure by practical exceptions and inconsistencies that engineering has to deal with but develops complex theoretical systems.
2.: In a slightly different vein is the work of abstraction and idealisation as one essential element of modern science (see p. 5). Here, pure is understood in the sense of ideal but not observable in the real world. The process of falling is separated from the dragging force produced by air. Hence, in a perfect vacuum, all objects (be it a feather or a ball of lead) fall in the same way, e.g. with the same acceleration. This meaning of purification is related to the first in so far as practical problems are disregarded.
3.: *Free of personal interests* The main constituent of the ethos of a researcher is that he or she should stand back form his or her personal beliefs, preferences and emotions. Seeking knowledge has to be performed by unbiased observation and disinterested logical reasoning. They will lose their connection to reality if observation and modelling are driven by beliefs and preferences (Weingart 2001, p. 51, 57, 59, 69).
  These three aspects are more related to philosophical argumentations on epistemology while both following aspects focus more on the social aspects of the organisation of research.
4.: *Free of practical purpose* In this sense, science is pure if it is not driven by purpose but has to be performed for its own sake, to gain knowledge, driven by curiosity. Pure science is not performed in order to be applied and to solve a practical problem. It does not have to be legitimised by its social relevance. This kind of orientation of science could be identified with basic science.
5.: *Free of political exertion of influence* In history, there are many examples where science has been misused for the justification of power. Especially, the dispute between science and religion to gain power during the Middle Ages has raised the awareness that freedom in research is a prerequisite for cognition (Weingart 2001, p. 76). Therefore, within the relation of science and society, there is a tricky balance between societal interests and funding (Weingart 2001, p. 78).

Within the discussion on technoscience, the different meanings are often combined or even mixed up. In some publications, the orientation of science towards application and social purpose alone qualifies science as technoscience: “Instead of seeking to humbly understand and explain a given nature, they now openly embrace the project of overhauling or transforming nature, of ‘Shaping the World Atom by Atom’” (Nordmann 2004, p. 59/60).

Firstly, I would like to point out that the idea that science is not only performed for its own sake or to gain knowledge but has to serve social purposes is closely related to the formation of modern science and was strongly claimed by Francis Bacon. Therefore, in order to discuss new developments within today’s scientific organisation, it seems to be useful not to restrict the meaning of technoscience to the fourth interpretation regarding the purity of science.

Secondly, a point I would like to raise in this context, is that the focus on practical purpose reduces the term technoscience to just another term for engineering and shifts the discussion towards the nexus of science, theory and engineering.

The obscurity of the amalgamation of representation and intervention denudes it to be pragmatism: while science is only interested in techniques in order to reveal theoretical hypotheses, engineering does not care for theory.^20^ From the engineering perspective, the job is done if it works, no matter how or why. This is exactly what Nordmann means, when he concludes that “…‘pure’ science is pure precisely because they invest a lot of analytical effort into the conceptual and technical separation of these two [representing and intervening] activities” but “[it] would be a moot exercise to take this pharmacological agent or to take the effected dilatation of the arteries and carefully tease apart what is due to human intervention and what to features of nature”^21^ (Nordmann 2010, p. 7). To sell a pharmaceutical, it is not necessary to explain how it works, it is important that it works and that it does not produce intolerable side effects. But if one would have progression in cognition, theory is necessary. And then it would be very helpful to know how the agent leads to the dilatation of the arteries. According to the hypothetico-deductive method, reliable hypotheses which can be tested can only be produced if there is a theoretical understanding. Otherwise trial and error, “tinkering”, or “bricolage” will tap into the dark and the next successful trial will be found just by chance.

### Control and domination

A further complex that is related to the term technoscience is domination and power. It is stated that, within technoscience, the subjection of nature has become a new quality (Stoff 2010, p. 119). Its inherent tendency to control is pervasively embracing society, personal relations, even the human body and might even alter the nature of human. This seems to be reached with genetic engineering and the cloning of mammals. The critique of this “scientification” and mechanisation of all aspects of daily life is a core subject in the discussion of technoscience (e.g. Weber 2003, p. 223). But this topic has a long tradition starting with Max Weber (Weingart 2003, p. 9), could be found in Jaspers (1949, p.127), might have had its peak in the 1960s (according to Degele 2002, p. 28), and has been discussed more recently by Jonas (1987), Habermas (2001), and Böhme (2002). The idea that science is performed to subdue nature was previously proclaimed by Bacon (Schäfer 1999) and is a core element of modern science. It has its origin in the ancient connotation of outwitting nature. Adorno and Horkheimer even argue that control and domination are inherent properties of reason and are related to the principle of abstraction and de-contextualisation (Adorno and Horkheimer 1991 [1944]; Horkheimer 1985 [1947]) (Adorno 1988 [1966]). This critique also can be found in the discourse on technoscience. There it is transformed into a question of purification of natural objects and the idea that the construction of purely epistemic objects is merely a way of trimming nature until it fits into the theory, leaving aside the ontological properties of the objects of investigation (Weber 2003). With the notion of the “molecularisation of life”, a similar property of modern science is critically denoted. This critic addresses the perception of nature as complex machinery mentioned above.

## Conclusion

If we look at the history of modern science, we could not find a specific point at which representation and intervention start to be indistinguishable. Instead, due to the fact that technique has been essential for modern science from its early beginning, modern science is characterised by a hybridisation of knowledge and intervention and by the production of their artificial objects of investigation. Moreover, the idea that knowledge is gained not for its own sake but to raise wealth and living conditions has been related to modern science from its beginning. The structural bracing of natural science, technological development and industrial exploitation of nature go back to the foundation of modern science. The answer to the question in the title of my paper—When does the co-evolution of technology and science overturn into technoscience?—would be: “Never or for ever”. Never, because representation and intervention still can be and even has to be distinguished in order to be able to perform cognition. For ever, which means here since the foundation of modern science itself, because the amalgamation of intervention and representation, of manipulation and observation of nature, of technology and science, are essential elements of modern science.

Furthermore, during the period since the foundation of modern science, the perception of the relation of nature and technology has changed radically. Since then, nature has been perceived in an abstract manner and as complex machinery which is organised by general laws. In contrast, the role of technology in science has changed continuously. The manipulation of nature in order to measure its properties has steadily increased until artificial things have been produced, such as laser beams, chemical compounds, elementary particles and Bose–Einstein-condensates. The STM and nanotechnology do not deserve a central position in this continuum of refinement of the use of technology within experimentation. If we perceive “the primacy of technology relative to science” (Forman 2007, p. 9) as the *differentia spezifica* between science and technoscience, it is questionable whether the term technoscience can be useful to reveal new developments within the organisation of science and the production of knowledge. Instead, the relation between observation and intervention is reformulated into the question: how far can “tinkering”, “bricolage” or engineering go, just by trial and error, without using theory?

From my perspective, the analytical value of the term technoscience is linked with the complex of control. This complex combines other aspects of technoscience. For instance, several meanings of “pure” coming together: abstraction and idealisation, practical purpose and political exertion. Therefore, I think that the complex of control is the most fruitful element of the term technoscience. Here, I see the innovative moment of the term. It allows the investigation of control and domination as an inherent mechanism of research which takes place in the way in which scientific objects are physically constructed. In addition, it reframes the question of the natural and the artificial which might lead to the monitoring of the shift of the unavailable (Barkhaus and Fleig 2002b).

Nevertheless, the use of techniques and the creation of artificial objects of investigation are not new phenomena. If the discussion on technoscience leads to new conclusions, the method of manipulating nature and the extent of that manipulation have to be considered. The specific characteristics of technoscientific objects have to be clarified as have the specific characteristics of the social organisation of technoscience and its performance. The precise role of technology in technoscience needs to be determined together with the concepts of nature on which technosciences rely. I think answers to these points could enhance the analytical value of the notion of technoscience and could help in understanding the new developments that science encounters.

## Footnotes

1 See e.g. (Baird and Shew 2002) and the workshop Imaging NanoSpace in 2005, Bielefeld (http://www.uni-bielefeld.de/(en)/ZIF/AG/2005/05-11-Nordmann.html).

2 While in the discussion on technoscience the term “representation” (Hacking 2008 [1983]) is more common than the term “observation”, the philosophical traditional base for the term is “observation” Stache (1958). In the philosophical tradition, the process of observation is not simply reduced to “seeing with the naked eye” but includes the relation and interaction of subject and object and the fact that observation is related to theory, especially in modern natural science. The term representation tries to bypass the problem of realism by leaving open the question of whether theory (representation) fits to reality (see e.g. Hacking 2008 [1983], p. 130ff). While Hacking is using the term representation in a very broad way (“When I speak of representations I first of all mean physical objects: figurines, statues, pictures, engravings, objects that are themselves to be examined, regarded. … Representations are external and public, be they the simplest sketch on a wall, or, when I stretch the word ‘representation’, the most sophisticated theory about electromagnetic, strong, weak, or gravitational forces” (Hacking 2008 [1983], p. 133)) it seems to be that Nordmann (2005) refers to the term “observation” in the cited paper. For my analysis the differentiation between representation and observation is not essential.

3 Nicolaus Copernicus' De revolutionibus orbium coelestium (On the Revolutions of the Heavenly Spheres) was published in 1543.

4 Due to his precise measurements, he could show in De nova stella (1573) that the dominant concept ‘celestial spheres’ are not consistent with his observations (http://en.wikipedia.org/wiki/Tycho_Brahe).

5 In Astronomia nova (1609), he published his theorem that planets are moving along ellipses with the sun at the focal point, which is known as the first Kepler law (http://en.wikipedia.org/wiki/Kepler#Astronomia_nova).

6 This project was later called the mathematisation of nature see Schäfer (1999, p. 100).

7 “Ad meritum et usus vitae” cited in (Schäfer 1999, p. 102), footnote 5.

8 Stokes differentiates science by the relation of understanding and use (Stokes 1997). From his perspective, both categories are goal oriented. But what is the goal of understanding if not the hope that one can use this understanding once for a mundane purpose? I am not fully convinced of his quadruple differentiation because between pure applied research (which is an idealisation and does not exist) and use inspired fundamental research (e.g. cancer research), there is no third thing. There is only a continuous shift in priorities of goals (understanding vs. use), or in other words a continuous transgression between science and technology.

9 The idea of progress related to cognition was not conceived by the ancient Greek philosophers. It is also a product of the age of modern science (Krohn 1989, p. 19).

10 Similar to material science, semiconductor physics was, from its beginning, closely intertwined with technological interventions and practical applications. Practical problems have initiated and inspired research questions—how to transform a MASER (Microwave Amplification by Stimulated Emission of Radiation) into a LASER—and effects observed in the laboratory (photoconductivity in 1873 by W. Smith) have been transferred to practical application (light meter 1875 by W. v. Siemens) (Wagemann 1992, p. 473).

11 There are similarities to the investigation of “new” elements of the periodic table in atomic physics. Similar to elemental particles, the objects of investigation (new elements) do not exist in nature but are produced before their characteristics are measured. Bernadette Bensaude-Vincent calls such entities “technoscientific objects” (2010).

12 In the discussion of technoscience this is also called “co-production of things and facts”.

13 Jutta Weber calls this the “ontological properties of objects of investigation” (Weber 2003, p. 231, transl. UF).

14 Nordmann argues: “The STM has become the symbol for nanoscience not because with its help we can see atoms, but we can move them intentionally” (Nordmann 2005, p. 7, transl. UF)*.* I agree with this statement. But I think the reason for this is not the how technology is used within science. It might be only a slight difference when I argue that the reason is that this instrument could be used to tell a story. It offers a link to the narrative “moving the very atom intentionally”. The STM plays an important role in constructing the nanotechnological myth that mankind (for the first time) is “shaping the world atom by atom”—Alfred Nordmann has contributed a lot to this perception of the role of these narratives. I think that there are additional aspects which gave the STM such a role. One is the fact that it produces pictures of surfaces which could be in colour and which are in a way familiar to us because they resemble pictures of landscapes (see e.g. Nordmann 2003; Lösch 2004). The other is related to the successful “proof of principle” by moving atoms by Don Eigler’s IBM-Picture.

15 The question whether nanotechnology is technoscience or marks a new relation between science and technology is a somewhat misleading question. The reason is that there are too many too different methods, research activities, technical applications and processes which are attributed to nanotechnology as if this were a meaningful assignment. It is an inadequate generalisation. It could be compared with the experiments to characterise a whole field of science (e.g. biology, chemistry, material research etc.) as being dangerous or harmless or helpful or what ever.

16 One can argue that the development of the atomic bomb and the ability to destroy all higher life on earth marks a new level of domination of man over nature. Usually this overstepping of limits is not used to characterise the age of technoscience and I do not see what one could gain analytically if one did.

17 The whole debate on human enhancement and transhumanity is an interesting phenomenon, which was related to robotics and artificial intelligence and has now found a new place in the field of nanotechnology. In this context I would like to emphasise that despite the fact that such discussions take place under the label of a certain technology, such as AI or nanotechnology, one cannot conclude that the aspects which are discussed are characteristics of this technology. Instead, the correlation between these issues and the technology has to be investigated and worked out.

18 The background to Kant’s project is the crisis of inductive cognition (Kant 1989 [1783]). Hume has shown that there is no logical bridge which leads from the singular result of an experiment to a general principle (Hume 1982 [1748]). Finally, this crisis led Popper to the conclusion that scientific principles can only be falsified but never verified (Popper 1963).

19 Theory as the recognition of essential phenomena (Schäfer 1998, p. 470; Schnädelbach 1998, p. 41).

20 As already mentioned in fn. 8, Stokes differentiates science by the relation of understanding and use. Both categories are—according to Stokes—goal oriented. If understanding is only performed for the sake of understanding, I think that “goal orientation” is a misleading category. If knowledge is to be striven for in order to be able to solve problems, understanding is only the indirect goal and problem solving the direct one. Further, this differentiation is misleading because between pure applied research (which does not exist) and use inspired fundamental research (e.g. cancer research), there is no third thing. There is only a continuous shift in priorities of goals (understanding vs. use) and thus a continuous transgression between science and technology.

21 Interestingly, the quote from Nordmann shows again the latent preoccupation with the question “what is natural?” and “what is artificial?” But here it is mixed with the question “what can I know about a subject?” The question “what belongs to nature?” or “what part of a process is natural and what is artificial?” is and was completely irrelevant for natural science. In modern science, elementary particles belong to nature together with the accelerator built to produce them. Whether something is produced by man or by processes which are not initiated or influenced by man (i.e. nature) is irrelevant for my understanding and thus is not a question of cognition.
